# Dielectrophoretic Traps for Efficient Bead and Cell Trapping and Formation of Aggregates of Controlled Size and Composition

**DOI:** 10.3389/fbioe.2022.910578

**Published:** 2022-07-14

**Authors:** Clémentine Lipp, Laure Koebel, Arnaud Bertsch, Michaël Gauthier, Aude Bolopion, Philippe Renaud

**Affiliations:** ^1^ Laboratory of Microsystems LMIS4, Ecole Polytechnique Fédérale de Lausanne (EPFL), Lausanne, Switzerland; ^2^ AS2M Department, CNRS, FEMTO-ST Institute, Université Bourgogne Franche-Comté, Besançon, France

**Keywords:** microfluidics, cell trapping, electroporation, cellular assemblies, dielectrophoreis (DEP)

## Abstract

We present a microfluidic dielectrophoretic-actuated system designed to trap chosen single-cell and form controlled cell aggregates. A novel method is proposed to characterize the efficiency of the dielectrophoretic trapping, considering the flow speed but also the heat generated by the traps as limiting criteria in cell-safe manipulation. Two original designs with different manufacturing processes are experimentally compared. The most efficient design is selected and the cell membrane integrity is monitored by fluorescence imaging to guarantee a safe-cell trapping. Design rules are suggested to adapt the traps to multiple-cells trapping and are experimentally validated as we formed aggregates of controlled size and composition with two different types of cells. We provide hereby a simple manufactured tool allowing the controlled manipulation of particles for the composition of multicellular assemblies.

## 1 Introduction

The necessity of developing tools able to manipulate single cells and analyse their behaviour at the single-object resolution is well established (Di Carlo et al., 2012; Dura et al., 2015; Abdulla et al., 2021). Unveiling cell heterogeneity by simultaneous temporal and single-cell resolution of cell response to external stimuli are difficult to obtain and often limited to one of the two with standard methods such as microscopy or flow cytometry. Microtechnologies offer the ability to manipulate objects at the single-cell resolution using different forces. In particular, dielectrophoresis (DEP) is a method of choice when a non-contact, active and versatile manipulation of cells or particles suspended in a liquid medium is required. Dielectrophoresis derives from a non-uniform electric field inducing a polarization of a particle. The direction of the force on the particle arising from this polarization is defined by design, but its magnitude can be tuned by the voltage applied to the electrodes and its positive (pDEP) or negative (nDEP) effect can be tuned in particular cases by the frequency. DEP has been widely used for separation of cells based on dielectric properties (Piacentini et al., 2011), but has also often been used to direct, trap and position single cells (Mittal et al., 2007; Godino et al., 2019; Punjiya et al., 2019). However, the presence of an electric field in a conductive medium can harm cells, and while many authors demonstrate the ability of their design to trap particles against a certain flow rate for a given voltage, very few assess the heat generation related to the traps (Seger-Sauli et al., 2005).

The formation of cell aggregates with controlled number and type of cells is crucial in the understanding of cancer invasion and development. For example the role of cancer associated fibroblasts in tumorigenicity is well known and the need for multicellular models based on co-culture to mimic the tumor environment was demonstrated (Labernadie et al., 2017; Lazzari et al., 2018), but there is a lack of tools to control the composition of multicellular assemblies down to the single cell level. In well plates, obtaining single cells using limiting dilution methods comes at the cost of a small portion only of usable wells due to Poisson distribution (Gross et al., 2015) and cell ratios are often determined based on volume and density to recreate heterogeneity (Bauleth-Ramos et al. (2020)). Dielectrophoresis has already been used as a tool to form aggregates of cells (Altomare et al., 2003; Menad et al., 2015; Cottet et al., 2019) and to trap and pair single cells in a controlled manner (Kirschbaum et al., 2008; Yoshimura et al., 2014) but has not yet, to our knowledge, been used to create heterogeneous aggregates with controlled number and type of cells.

In this work, we propose an original electrode design offering an efficient three-dimensional dielectrophoretic trap for single cells together with a distribution system. We propose two easy to fabricate configurations, compare their trapping efficiency and assess their heat generation. The most efficient configuration is selected and the limits of voltage necessary to avoid cell membrane electroporation is determined. The design rules to accommodate more cells in higher channels is defined. We demonstrate the capability of the presented system to direct and arrange cells in a controlled manner by forming multicellular assemblies of predetermined size and composition.

The device we propose is easy to fabricate and offers efficient three dimensional trapping capabilities with simple coplanar electrodes and is thus accessible to researchers disposing of standard equipment. The distribution system allows a full control on the positioning of the arriving cell and unlocks for the first time the ability to form heterogeneous assemblies of cells with pre-determined number and type of cells using DEP. This feature is key in research domains focusing on cancer stem cells and the corresponding drug development strategies (Ishiguro et al., 2017), but finds also applications in studies of cellular aggregates that mimic the cancer micro-environment (Ham et al. (2016)). While electroporation of the created assembly was avoided in this study, it can be exploited and is readily available in drug development or applications where cell transfection is desired (Chopinet et al. (2012)). Furthermore, domains studying cell adhesion and receptor-ligand interactions will benefit from this device’s ability to form pairs of beads and cells (Dura et al. (2015)). We demonstrated here these features using an array of four traps, but the number of traps can be easily scaled up by lateral repetitions of the trap units as well as repetitions of pairs of electrodes along the length of the channel.

## 2 Material and Methods

### 2.1 Microfabrication

**FIGURE 1:**
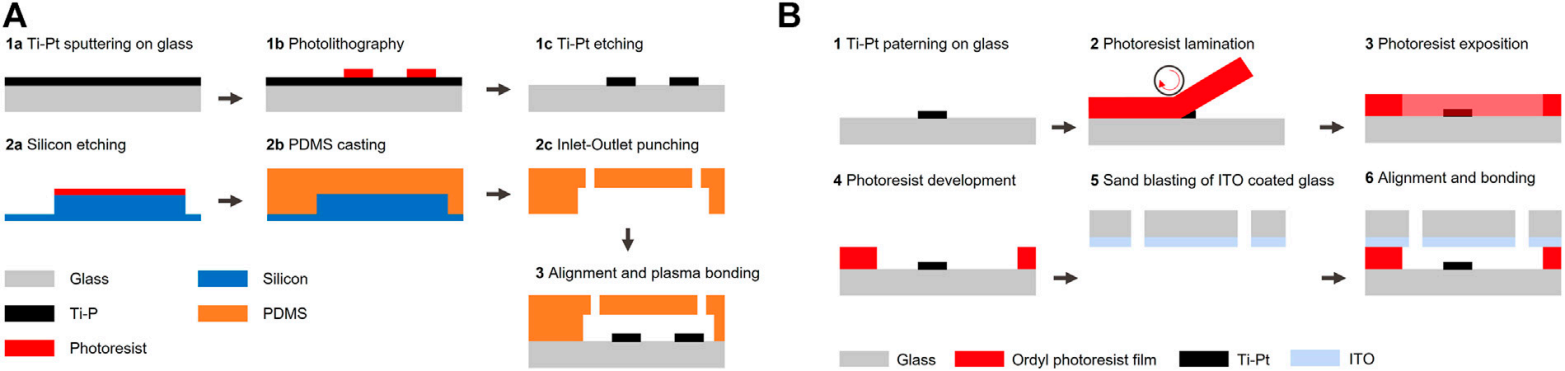
Process for the fabrication of the chips. **(A)** The fabrication of coplanar configuration chips starts by the patterning of Ti-Pt electrodes on a glass substrate (wafer 1). PDMS is cast on a silicon mold, punched, aligned and permanently bonded to the glass chips (wafer 2). **(B)** The fabrication of facing configuration chips starts by the patterning of electrodes on a glass wafer and is followed by the lamination and patterning of a photosensitive film to define the (wafer 1). The cap wafer coated with ITO (wafer 2) is then aligned and bonded to the bottom chip.

The coplanar electrodes chips were fabricated using the following process: borofloat wafers were first cleaned using a piranha solution. The metal layer was deposited by sputtering 20 nm of titanium and 200 nm of platinum (SPIDER, Pfeiffer) (Figure 1A step 1a). Photoresist (AZ 1512 HS, MicroChemicals) was spincoated, exposed by direct laser writing (MLA150, Heidelberg Instruments) and developed (ACS200, Süss) (Figure 1A step 1b). Unprotected metal was etched using ion beam etching (IBE350, Veeco Nexus) and chips were diced (DAD321, Disco) (Figure 1A step 1c). The PDMS microfluidic channels were fabricated using a process described elsewhere (Cottet et al. (2017)). Shortly, the PDMS master mold was fabricated by deep reactive ion etching of a silicon wafer (AMS200, Alcatel) (Figure 1A step 2a). PDMS was molded, cured, punched, precisely aligned and permanently bonded to the glass chips patterned with electrodes using a mask aligner (MJB4, Süss) (Figure 1A steps 2b-3).

The facing electrodes chips were fabricated following a process described previously (Vulto et al., 2005), by first patterning electrodes as described in the previous paragraph (Figure 1A steps 1a-1c). A photosensitive adhesive film was laminated (Ordyl®SY320, 20 *μ*m thickness) (Figure 1B step 2), exposed through a mask using a mask aligner (MJB4, Süss) with a postbaking step of 1 min at 85°C, developed (Ordyl ®XFB) and rinsed (Ordyl ®Rinse) (Figure 1B step 3–4). The fluidic inlets and outlets of the capping borofloat wafer were fabricated by sandblasting (IcoFlex Sàrl, Switzerland) and 200 nm of ITO was then sputtered (SPIDER600, Pfeiffer) (Figure 1B step 5). Both wafers were then aligned (MA6/BA6, Süss) and bonded (SB6, Süss) by applying a pressure of 425 kPa for 30 min at 90°C followed by a curing step at 150°C for 2 h (Figure 1B step 6). Connections between the top and bottom electrode were made by placing a chip on a heating plate at 90°C and introducing low temperature solder (The Indium Corporation of America) inside a microfluidic channel designed for this application and passing on top of the desired bottom electrode track.

### 2.2 Materials

5 *μ*m in diameter polystyrene beads were purchased from Sigma- Aldrich and suspended in a working solution composed of 10% in volume phosphate buffered saline (PBS) (Gibco) and 90% deionized water at a concentration of 5 ⋅ 10^5^ beads/ml. The surface of polystyrene beads was left uncoated and the particles are thus natively negatively charged in buffers with physiological pH. The use of AC electric fields however discards any net movement due to electrostatic forces.

### 2.3 Cell Culture

Jurkat and Colo205 cell lines (ATCC) were cultured in RPMI 1640 supplemented with 10% of fetal bovine serum (FBS) and 1% Penicillin-Streptomycin at 37°C in 5% CO_2_ atmosphere. Staining of Colo205 was performed by incubating the cells in PBS with 4 *μ*m Calcein UltraBlue™ AM (Cayman Chemical) for 1 h. Staining of Jurkat cells was done by incubation in PBS with 1 *μ*m Calcein AM (Invitrogen™) for 1 h. The working solution is composed of 40% RPMI and 60% deionized water. The solution is compensated for osmolarity by the addition of dextrose (Sigma-Aldrich) and cleaned through a 0.22 *μ*m filter. Jurkat and Colo205 were both resuspended in the working solution and passed through a 40 *μ*m cell strainer before the experiment. All reagents are from Gibco unless specified.

### 2.4 Chip Operation

Measurement of the current and phase was performed in a solution of 10% PBS and 90% deionized water by applying an AC signal between 1 and 10 V amplitude to the trapping electrodes at a frequency of 100 kHz using an HF2TA current amplifier connected to a HF2LI Lock-In amplifier (Zurich Instruments).

The PDMS chip was primed with Pierce™ Protein-Free (PBS) Blocking Buffer during 1 h to prevent cells from adhering to the surfaces. The cells or beads were placed in a chromatography vial connected to the punched PDMS by a 360 *μ*m outer diameter PEEK tubing (Idex). Pressure was applied to the vials using Fluigent Flow-EZ pressure controllers. The chip was mounted on and electrically connected to a custom PCB placed on the stage of a Leica DMI3000 B inverted microscope and observed using a uEye (IDS) camera. All the electric signals needed to control the positions of the particles are sent through a home made PCB creating the multiplication of an AC signal at 100 kHz and different DC signals whose amplitudes are controlled by the computer with an adapted *C++* program through an analog output generator (Mccdaq USB-3100).

### 2.5 COMSOL Simulations

Modeling of the electric field and DEP force direction and magnitude were done using COMSOL Multiphysics 5.6 with the Electric Currents and Creeping Flow modules. The medium electrical conductivity and relative permittivity were set respectively to 0.16 S/m and 78. The fluid flow at the entrance of the channel was set to 700 *μ*m/s. A sinusoidal electric potential of 10 V amplitude and 100 kHz frequency was applied to the trapping electrode and the potential of the counter electrode was set to zero.

## 3 Results and Discussion

### 3.1 Concept

**FIGURE 2:**
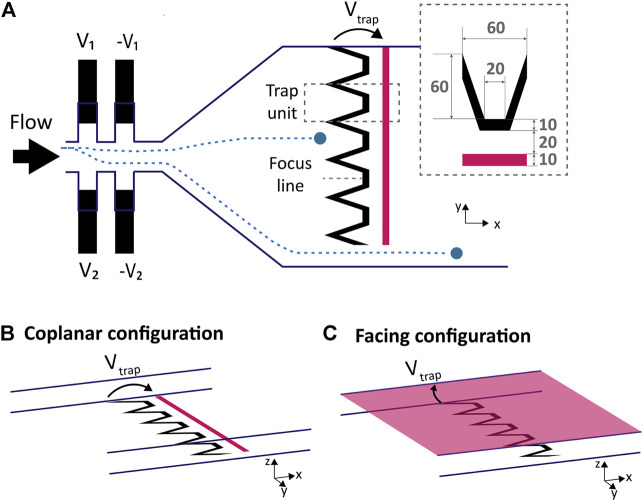
Deviation system, electrodes design and configuration. **(A)** Top view scheme of microfluidics and electrodes: the deviation system is based on liquid electrodes placed on each side of the channel, upstream of the trapping electrodes. The ratio of the voltages applied on each side (*V*_1_/*V*_2_) defines the lateral position of the particles (Demierre et al., 2007), directing it towards a defined trap unit. Two examples of particles trajectories are illustrated in blue. The main electrode is composed of trap units placed next to each other and of a counter electrode highlighted in pink. The trapping electrodes are not covering the whole channel width so the unwanted cells can bypass the traps. The dimensions in *μ*m of a trap unit are displayed in the inset. **(B)** In this coplanar configuration the counter electrode is a straight line placed downstream of the main electrode on the same plane. **(C)** The counter electrode of the facing configuration covers the cap of the microfluidic channel.

We propose a microfluidic system capable of performing in-flow cell selection, sorting and trapping using non-contact DEP actuation. The system is presented in Figure 2A is composed of an upstream DEP actuated deviation system that laterally focuses the incoming particles or cells on specific flow lines so that they can be collected by the desired downstream DEP trap. The DEP traps are actuated by applying a potential across two electrodes: the upstream main electrode is designed with repetitions of funnel-shaped trap units and the counter electrode is either placed in a coplanar or facing configuration. Each trap unit is 60 *μ*m in width and capable of stopping a single cell or particle in flow by balancing the drag force and creating a single position of equilibrium upstream of the electrodes. The slanted parts of the trap unit finely focus the particles towards the center of the traps indicated by the focus line in Figure 2A. The number of traps can be adjusted to the width of the channel by lateral repetitions of the funnel-shaped trap unit. A bypass area is also provided next to the trapping electrodes such that unwanted cells can be directed to bypass the traps and leave the chamber.

#### 3.1.1 Deviation System

In order to control the trajectory of the incoming particles and guide them towards the desired trap unit, a deviation system using six pairs of liquid electrodes similar to the one presented by Demierre et al. (2007) was placed upstream of the trapping chamber. Only two pairs of liquid electrodes are represented on the left of Figure 2A. Liquid electrodes are coplanar electrodes placed in a lateral recess from the main channel: this configuration guides the electric field and generate a vertical equipotential surface at the entrance of the main channel, hence the name “liquid electrodes”. Two sets of liquid electrodes combs are placed laterally on each side of the main channel and a voltage is applied to each set, with a phase shift of 180° to neighbouring electrodes. Each set of liquid electrodes generate a lateral DEP force uniform along the height of the channel pushing the particles away from them. The ratio of voltage applied to each side *V*_1_/*V*_2_ determines the equilibrium position along the *y* axis in the main channel, focusing the randomly distributed incoming particles towards a defined lateral position and deviating them towards the trap units, or to the bypass. As described by Demierre et al. (2007), the total force exerted on the particles is the sum of the forces exerted by each comb of electrodes and the particles are focused where these forces cancel each other: it is thus non dependent on the particle or cell size nor position before entering the deviation zone. At low fluid velocity, the limit in the focusing is due to particles collision and diffusion, which is compensated by the slanted part of the traps placed downstream, collecting particle streams covering 60 μm in width. The focusing nevertheless has a size dependent limit on the channel sides: the particle’s center cannot be focused closer to the wall than the particle’s radius due to straightforward reasons.

#### 3.1.2 Counter Electrode Configuration and Simulations

**FIGURE 3:**
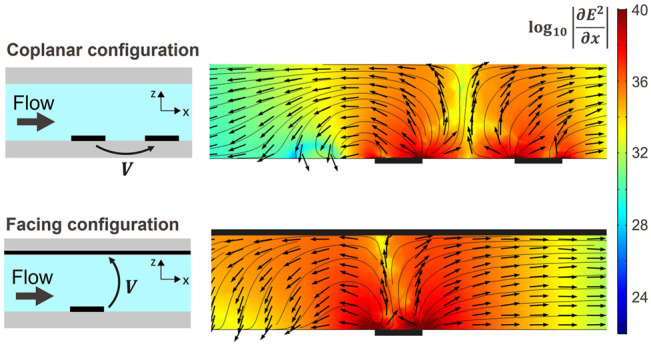
COMSOL ™ simulation along a *xz* plane in the center of the trap as indicated by the focus line in Figure 2A) for both configurations and for a voltage amplitude of 10 V. The colour indicates the magnitude of $\frac{\partial E^{2}}{\partial x}$ , which is the electric field dependent factor that modulates the DEP force, while the arrows and streamlines indicate the direction of the nDEP force along the *xz* plane. The electrodes are drawn on top of the simulations for visualization. Both electrodes configuration generate a force pushing against the flow and towards the bottom of the channel upstream of the main electrode. As the *y* component of the force points towards the center of a trapping unit thanks to the slanted part of the electrodes (see Supplementary Figure S1), both configurations generate a three dimensional trap against the flow.

Two easy to fabricate configurations of counter electrodes were implemented and studied: a coplanar counter electrode which consists in a straight line electrode placed downstream of the trapping electrode as shown in Figure 2B, whereas for the facing counter-electrode, a conductive and transparent ITO layer is provided on the glass plate used to close the channels on their upper part as illustrated in Figure 2C. Both configurations are popular and extensively used to manipulate cells using DEP in microfluidic chips (Ho et al. (2013); Li and Bashir (2002); Morgan et al. (1999); Takahashi and Miyata (2020)). Other electrodes configurations that are known to create single cell traps comprise 3D electrodes (Keim et al., 2019) and strip electrodes (Seger et al., 2004), but such configurations are less accessible in terms of fabrication. In order to assess the three dimensional behaviour of the traps, both designs were simulated using COMSOL ^(^™^)^. Figure 3 illustrates the results of the simulation along the focus line indicated in Figure 2A: the colour indicates the magnitude of $\frac{\partial E^{2}}{\partial x}$ , which is the electric field dependent factor that modulates the DEP force while the arrows of normalized size indicate the direction of the nDEP force along the *x* and *z* axis. Electrode locations are indicated on the results of simulations by black rectangles. Simulations indicate that both configurations can be used for the trapping of particles in three dimensions: the *y* component of the force directs the particles toward a single *xz* plane of equilibrium in the middle of each trapping unit (Supplementary Figure S1) while the *z* component pushes the particles towards the bottom of the channel. The *x* component of the DEP force counteracts the drag force. Its value, controlled by the applied voltage, determines the equilibrium position of the particles along the *x* direction. For the same voltage applied, the facing electrodes configuration generates a larger DEP force in the *x* direction compared to the coplanar configuration.

To test these two designs, chips with electrodes placed in both configurations and aligned to microfluidic channels with *μ*m precision can be manufactured using standard cleanroom equipment with a few steps only. The fabrication process for both designs is detailed in the Materials and Methods section and illustrated in Figures 1A,B. Experiments are presented in the next section.

### 3.2 Single Object Trapping

#### 3.2.1 Bead Trapping Efficiency

DEP can damage cells as it can induce a local rise in temperature. Indeed the presence of an electric field in a conductive media induces Joule heating. This effect can be mitigated by reducing the conductivity of the medium and correcting for osmolarity with the addition of dextrose or sucrose, but prolonged exposition of cells to such diluted media can alter their function and health (Hyler et al. (2021)). As both electrodes configurations presented here create a three dimensional DEP trap against the flow, we selected the most efficient configuration by measuring the magnitude of the DEP force against the flow for both configurations and evaluated in both cases the induced temperature rise. The expression of the time averaged DEP force exerted on a spherical particle in a non uniform electric field is given as follows:

$$F_{\text{DEP}}=2\pi \epsilon_{m}R^{3}Re\left[K\left(\omega \right)\right]{\nabla E}_{\text{rms}}^{2}$$ (1)

Where *R* is the radius of the particle, *ϵ*_*m*_ is the fluid permittivity, *Re* [*K*(*ω*)] is the real part of the Clausius-Mossotti factor and **E**_rms_ is the root mean square (rms) of the electric field. For a homogeneous spherical particle, the Clausius-Mossotti factor is given by the following formula:

$$K\left(\omega \right)=\frac{\epsilon_{p}^{*}-\epsilon_{m}^{*}}{\epsilon_{p}^{*}+2\epsilon_{m}^{*}}$$ (2)

With $\epsilon_{p}^{*}$ the complex permittivity of the particle and $\epsilon_{m}^{*}$ the complex permittivity of the medium, which are both frequency dependent. The sign of the Clausius-Mossotti defines the regime of the DEP force, negative or positive, resulting in particles being respectively repulsed from or attracted to the high electric field regions. In this work, we operate at 100 kHz in the negative DEP regime and the real part of the Clausius-Mossotti factor has a value of −0.5 for all medium and particle or cell conditions.In order for a particle to be trapped, the projection of the DEP force on the axis of the flow direction (*x* in the present case) has to balance the viscous drag force exerted by the flow (Voldman et al. (2001); Rosenthal and Voldman (2005)):

$$6\pi \eta R\left(6v_{\text{mean}}\frac{F^{*}z_{p}}{h}\right)=2\pi \epsilon_{m}R^{3}Re\left[K\left(\omega \right)\right]\frac{\partial E_{\text{rms}}^{2}}{\partial x}$$ (3)

Where *η* is the fluid viscosity, *v*_mean_ is the mean fluid velocity, *h* is the height of the channel, F* is a factor accounting for the wall effect, *z*_*p*_ is the height of the particle.

The heat flow generated by electrodes matches the electrical power input in the system and the average temperature around the DEP electrodes was shown to depend on the real part of the electrical power (Ramos et al., 1998; Seger-Sauli et al., 2005) which is defined as:

$$P=V_{\text{rms}}I_{\text{rms}}\cos\left(\theta \right)=\frac{V_{\text{rms}}^{2}}{Z}\cos\left(\theta \right)$$ (4)

With *V*_rms_ the rms voltage applied to the electrodes, *I*_rms_ the rms electrical current, *θ* the phase shift between the current and voltage and *Z* the norm of the electrical impedance.

Since the temperature is directly proportional to the electrical power, this latter can be used as an indicator of electrodes trapping efficiency when comparing designs of similar size. Following Equation (3), the DEP force developed by a trap at steady state and at a defined position is directly proportional to the velocity of the fluid flow dragging the particle. We thus measured the maximum DEP force developed by each configuration by immobilizing a polystyrene particle in a trap at low fluid flow and increasing the flow until the bead is released. We measured the particle velocity at release, which is directly proportional to the maximum DEP force the trap can develop in the *x* direction at the edge of the electrode as a function of applied voltage and developed power. This latter is obtained by multiplying the applied voltage by the measured current at each condition following Eq. 4.

From Eq. 3, the mean velocity of the fluid can be expressed as:

$$v_{\text{mean}}=\frac{h}{18\eta F^{*}z_{p}}\epsilon_{m}R^{2}Re\left[K\left(\omega \right)\right]\frac{\partial E_{\text{rms}}^{2}}{\partial x}$$ (5)

Since the potential distribution in space is a direct function of the voltage *V* applied to the electrodes, we can derive that (see Supplementary Information) $\nabla E_{\text{rms}}^{2}=\alpha \left(x,y,z\right)V^{2}$ , where ***α*** is a function of space. Eq. 5 can thus be re-written as:

$$v_{\text{mean}}=\frac{h}{18\eta F^{*}z_{p}}\epsilon_{m}R^{2}Re\left[K\left(\omega \right)\right]\alpha_{x}V^{2}$$ (6)

Introducing the electrical power into this equation from Eq. 4 we obtain:

$$v_{\text{mean}}=\frac{h}{9\eta F^{*}z_{p}\cos\left(\theta \right)}\epsilon_{m}R^{2}Re\left[K\left(\omega \right)\right]\alpha_{x}ZP$$ (7)

With *α*_*x*_ the *x* component of ***α*** (*x*, *y*, *z*) described above and taken at the edge of the electrode, this equation indicates that the efficiency of trapping of a given design and configuration of electrodes depends on the multiplication of *α*_*x*_ and *Z*.

**FIGURE 4:**
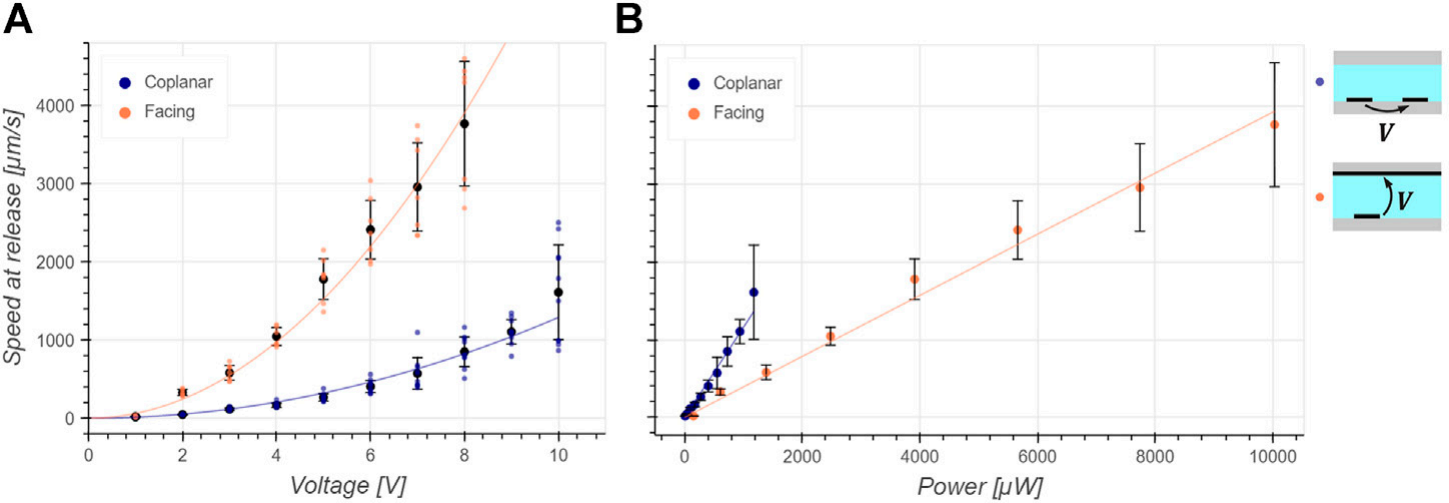
Characterization of the trapping efficiency: 5 *μ*m in diameter polystyrene beads are trapped for a given voltage and power and the flow is increased until the bead takes off and its speed is measured. The black dots and bars are the mean and standard deviation respectively and the continuous line is the fit. **(A)** The speed at release follows a squared dependency on the applied voltage as predicted by Eq. 6. As expected from simulations in Figure 3, the coplanar configuration develops a smaller force for a given applied voltage compared to the facing configuration. **(B)** The speed at release depends linearly on the developed power as expected from Eq. 7. It can be deduced that the coplanar design is more efficient at trapping in this case as it can develop a larger *F*_DEP,*x*_ for a given power dissipated than the facing configuration.

The current and phase were measured for both configurations and for voltage amplitudes between 1 and 10 V. The real part of the electrical power, responsible for Joule heating, was calculated as of Eq. 4. The mean and standard deviation phase was measured to be 11.2° and 1.4° respectively for the coplanar configuration and 8.9° and 1.5° respectively for the facing configuration, the real part of the power accounted for more than 97% of the apparent power thus indicating a mainly resistive load. Figure 4A is a plot of the measured speed at release for 5 *μ*m in diameter polystyrene particles as a function of applied voltage amplitude for both facing and coplanar configurations. The dependency of the velocity at release is quadratic on the voltage in accordance with Eq. 6. As predicted by the simulations and Figure 3, the facing configuration generates a larger $\frac{\partial E_{\text{rms}}^{2}}{\partial x}$ for a given voltage and the velocity of the particle at release is larger in this configuration. Figure 4B shows the velocity at release as a function of electric active power. It results that the velocity at release depends linearly on the power as predicted from Eq. 7. It appears that the larger gradient factor *α*_*x*_ of the facing design does not compensate the smaller impedance of this design, resulting in more heat dissipated for a given DEP force than the coplanar design and thus a larger increase in temperature. The coplanar design was thus selected for the following study of cell trapping. However, the facing electrode configuration is suggested for applications where flow speed is the main criteria and heat generation is not a limitation since it generates a larger DEP force for a given voltage.

#### 3.2.2 Single Cell Trapping

**FIGURE 5:**
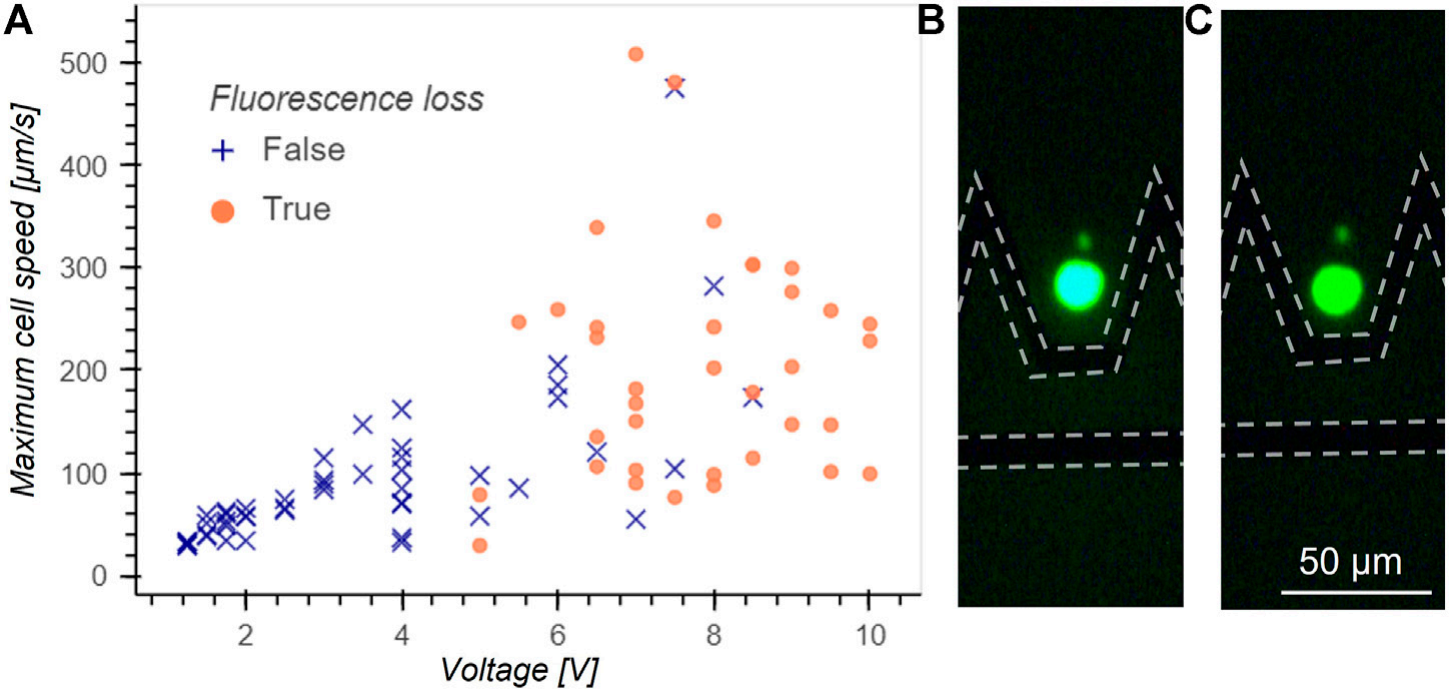
**(A)** Maximum cell speed at release without fluorescence loss as a function of voltage applied for Jurkat cells. Blue crosses indicate no fluorescence loss events and orange circles indicate a loss of fluorescence. A clear threshold under which cells never lose fluorescence is found at 5 V and determines the limit in voltage to avoid cell damage. The phenomenon behind the fluorescence loss is believed to be electroporation. **(B)** Fluorescence picture of a cell arriving in the trap before fluorescence loss. The position of electrodes is highlighted by a dotted line. **(C)** Fluorescent picture of the same cell after fluorescence loss.

Electroporation can damage the cells when trapped using DEP. Electroporation takes place when the potential difference accross the cell membrane exceeds a threshold value, inducing pores in the membrane. This phenomenon is not necessarily lethal for the cells and is widely exploited to introduce genetic material inside the cells. However electroporation is not desired in manipulation applications and to avoid any damage to the cells we experimentally determined the applied voltage limit to avoid electroporation conditions. Jurkat cells were loaded with Calcein AM to visualize pore formation: as calcein is a volatile fluorescent molecule, it quickly diffuses out of the cell in case of pore formation in the membrane. The maximum velocity for trapping without electroporation as a function of voltage amplitude is measured and reported in Figure 5A. In case of fluorescence loss, the voltage was immediately turned off to release the cell and measure its speed. Events where a leak of calcein was observed are indicated by an orange circle, whereas events without fluorescence loss are indicated by a blue cross. A clear threshold under which no fluorescence loss is observed is found for a voltage of 5 V amplitude and determines the voltage operation limits to avoid cell damage. The large variation in speed at release can be explained by variations in cell size. Figure 5B is a picture of a cell arriving in the trap and Figure 5C shows the cell after fluorescence loss.

Typical transmembrane potential threshold above which pores appear have been reported between 0.25 and 1 V (Escoffre et al. (2011)). Schwan equation relates the transmembrane potential ΔΦ_*m*_ to the external alternating electric field *E* with angular frequency *ω*:

$$\Delta \Phi_{m}=\frac{1.5RE\cos\left(\varphi \right)}{{\left[1+{\left(\omega \tau \right)}^{2}\right]}^{1/2}}$$ (8)

Given *τ* = *RC*_*mem*_ (*ρ*_int_ + *ρ*_*ext*_/2) with *C*_*mem*_ the membrane capacitance, *ρ*_int_ and *ρ*_*ext*_ the resistivity of respectively the internal and external fluid and φ the angle between the electric field lines and a line drawn from the center of the cell to the considered point of interest on the cell membrane. The critical value for transmembrane potential corresponds to a range of electric field between 2.8⋅10^4^ and 11.2⋅10^4^ V/m using membrane properties values from Reichle et al. (2000). Such values of electric field are found at the edge of the electrode and for a height of 5 *μ*m in simulations for applied voltage amplitude ranging between 2.3 and 9.3 V and comprising the experimentally found threshold, consolidating the hypothesis of fluorescence leakage due to electroporation. The maximum voltage for cells manipulation without electroporation was thus set to 4 V amplitude. Larger voltages may be used without electroporating the cells as long as the flow drag force is limited and does not bring the cell in the critical electric field close to the electrode edge.

### 3.3 Multiple Cells Trapping

The presented cell DEP traps with controlled deviation system can be used to trap multiple particles of different types in a single trap. Indeed the formation of aggregate of controlled composition and size can be of high interest to study the growth of tumors in their environment composed of different cell types. For applications that require to stop more than 1 cell, the trap and microfluidic channel need to be scaled up.

#### 3.3.1 Scaling Rules

**FIGURE 6:**
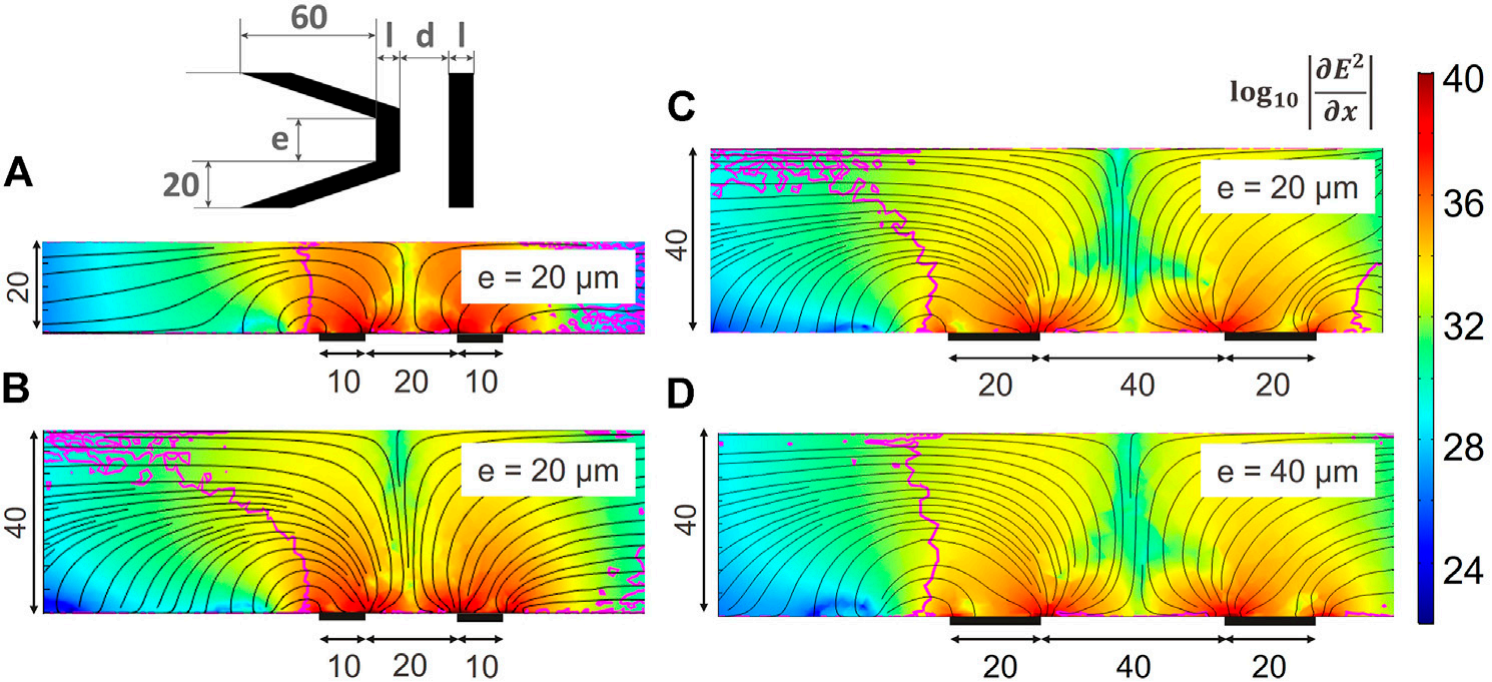
Effect of the geometry and channel height on the three dimensional trapping behaviour of coplanar electrodes. The colour indicates the magnitude of the *x* component of the gradient of the electric field squared, the black streamline indicate the direction of the DEP force and the magenta contour line separates regions with downward *F*_DEP,*z*_ on the left hand side, from upwards *F*_DEP,*z*_. All dimensions are in *μ*m. A vertical contour line is desirable to conserve a three dimensional trapping and avoid that particles found in the higher part of the channel flow over the trap without being stopped. **(A)** Original design as described in Figure 2 in a channel of 20 *μ*m height. **(B)** Original design in a higher channel: the three dimensional trapping behaviour is lost. **(C)** Distance between the electrodes and electrode width are scaled accordingly to the channel height. The contour line is still bent. **(D)** Homothetic scaling of the original design: the vertical trapping behaviour is recovered.

Different parameters were studied to understand their impact on the three-dimensional trapping property for an increased channel height. We defined the following parameters: *d* is the distance between the electrodes, *l* is the depth of the electrodes, *h* is the height of the channel and *e* is the width of the parallel part of the main electrode as illustrated on the inset of Figure 6. The angle of the slanted part was kept constant as well as the trap depth. Figure 6 is the result of COMSOL ™ simulations with the color indicating $log_{10}\left(\frac{\partial E^{2}}{\partial x}\right)$ , the black streamlines indicate the direction of the DEP vectors for different geometries. The magenta line indicates the contour of a null *z* component of the DEP force and thus separates the regions with upwards and downwards DEP force. A vertical contour defines a stable trap for a wide range of sizes and positions in the channel (Rosenthal and Voldman (2005)). Indeed, particles with a center of mass in the region with upward DEP force will be pushed upwards where the DEP force counteracting the flow is weaker, and therefore leave the trap. It is especially a problem when trapping multiple objects where the chance of finding an object higher in the channel is larger. As shown in the previous discussion, the standard design has a vertical contour and all particles coming in the traps will be pushed down to the floor. However, Figure 6B shows that this property is lost when keeping the same electrode geometry and increasing the channels height *h*. The scaling of the electrodes depth *l* and distance between them *d* is not enough to recover the vertical contour as seen on Figure 6C. A homothetic scaling is necessary to obtain the property of DEP force pushing down to a single equilibrium position along the whole trap height and to create a compact aggregate, as shown in Figure 6D.

#### 3.3.2 Formation of Aggregates of Controlled Size and Composition

**FIGURE 7:**
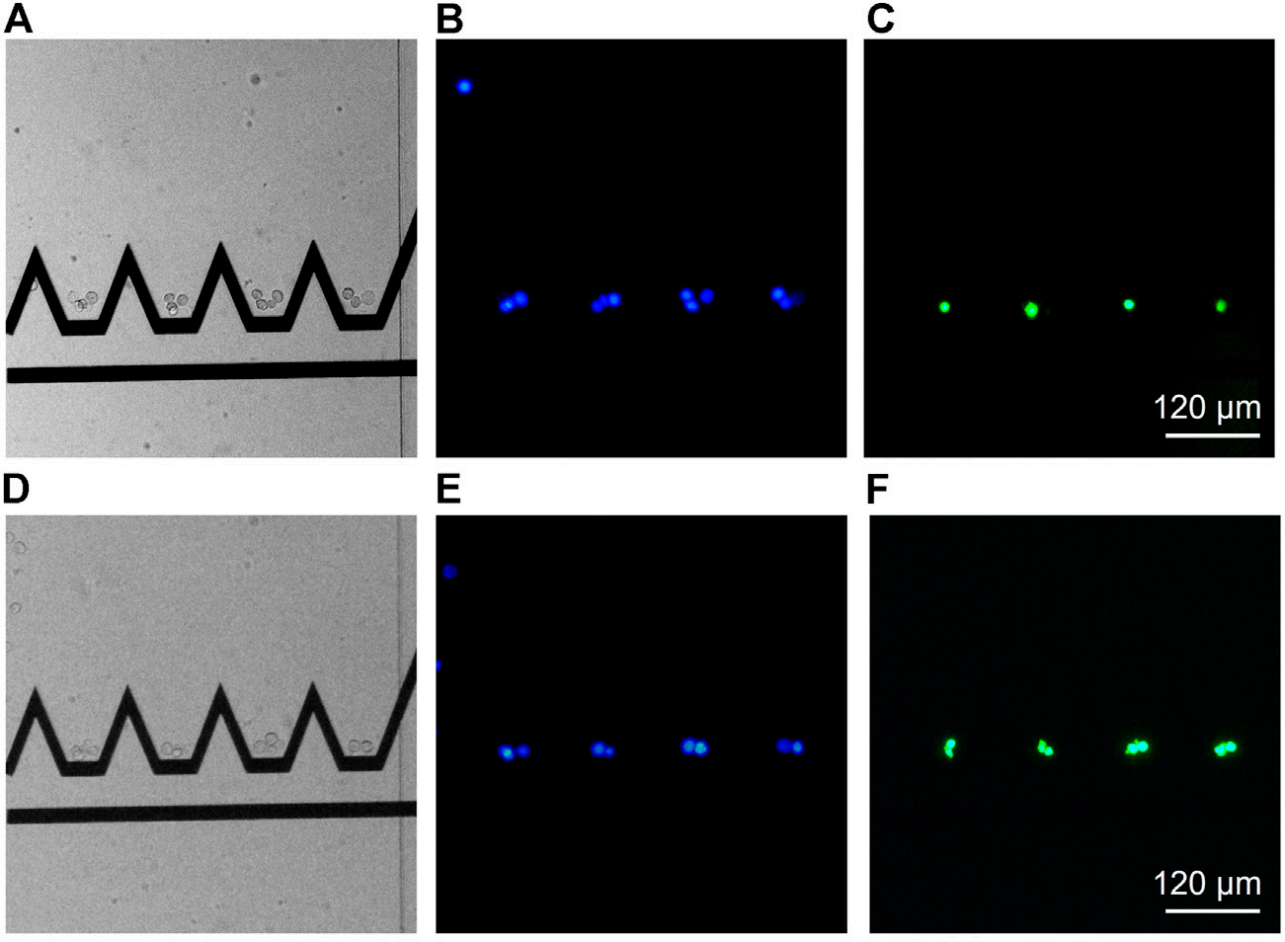
Proof of concept of the ability of the presented system to create aggregates of controlled size and composition. A homothetic scaling of the original design was done to accommodate more cells in the traps in a microfluidic channel of 40 *μ*m height. Four trapping units are placed in parallel and a bypass is present on the left to discard unwanted cells. The left images are brightfield images, the middle images show Colo205 cells stained with blue calcein and the right images show Jurkat cells stained with green calcein. **(A–C)**: aggregate of 4 cells composed of three Colo205 (blue) and one Jurkat (green). **(D–F)**: aggregate composed of two Colo205 (blue) and two Jurkat (green). As the aggregates rotate under the effect of the flow, the pictures could not be superimposed.

We demonstrate here the ability of the presented system to create cell aggregates of controlled size and composition. The single cell design was scaled up as in Figure 6D to accommodate more cells in a channel height of 40 *μ*m. Four parallel trapping units were placed next to each other and a bypass with no electrode was left next to the trapping units in order to discard unwanted cells. Two inlets were used sequentially to perfuse solutions with respectively Colo205 cells stained with blue calcein AM, and Jurkat cells stained with green calcein AM. The upstream deviation system was used to direct the incoming cells toward the desired trapping unit to create aggregates of 4 cells composed of three Colo205 and one Jurkat (Figures 7A–C) as well as two Colo205 and two Jurkat (Figures 7D–F). The calcein staining was used to both identify the cell type as well as to ensure cell viability. The aggregates could be held inside the traps up to 5 minutes without witnessing any leakage of the dye indicating membrane poration.

## 4 Conclusion

This study first proposes a design of electrodes capable of generating three-dimensional single-object DEP traps in two different configurations of counter electrodes. This design offers the possibility of increasing the number of traps actuated simultaneously and we demonstrate the use of a DEP deviation system to direct the particles towards the desired traps in a controlled fashion. We proposed a simple method for the evaluation of DEP traps efficiency for designs comparison. The method evaluates the maximum DEP force the trap is capable of developing to counteract the drag force and assesses its relationship to the power, mainly dissipated in Joule heating, necessary to generate it. We used this method to compare the two types of electrodes configurations and deduce that coplanar electrodes configuration is more efficient than the facing electrodes configuration. The coplanar configuration was used to trap Jurkat cells and the voltage limit to avoid electroporation was experimentally assessed. The scaling rules were defined to follow changes in channel height and a scaled design was used to create aggregates of 4 cells with controlled number and type of cells. We defined the voltage threshold for electroporation events, and when performed in a controlled way, a pulse of high voltage once cells are trapped can offer the possibility of controlled electroporation on specific cells or aggregates for transfection or electrofusion applications.

We believe that the proposed method to evaluate the efficiency of DEP traps based on power dissipation is crucial when trapping cells. The resulting coplanar configuration chip with deviation system offers a versatile tool for single cells and cell aggregates manipulation and studies and we believe will be useful to study biological interactions between cells and cellular assemblies.

Future development of the device will comprise a scale up in the number of traps and the full automation of the system using computer vision or impedance based feedback. Additional deviation system at the outlet of the interaction chamber will enable the sorting of the created assemblies. Additionally, placing a chamber comprising an array of hydrodynamic traps such as those presented by Bourn et al. (2020) to immobilise the formed assemblies downstream of the interaction chamber will allow a change of medium for long term on-chip culture and their longitudinal studies.

## Data Availability

The original contributions presented in the study are included in the article/Supplementary Material, further inquiries can be directed to the corresponding author.
