# Supplementary material for: Dielectrophoretic Traps for Efficient Bead and Cell Trapping and Formation of Aggregates of Controlled Size and Composition
Source: Front Bioeng Biotechnol. 2022 Jul 14;10:910578. doi: 10.3389/fbioe.2022.910578 (PMC9333130; doi:10.3389/fbioe.2022.910578)
Supplement: Supplementary file 1 [file Presentation1.pdf]

Isosurfaces of E^2

Isosurfaces of E^2 for the coplanar (A) and facing (B) configurations. The direction of the nDEP force is perpendicular to the isosurfaces and directed towards the isosurface of smaller value. The funnel shaped surfaces indicate that the force is laterally directed towards the center of the trap, points towards the bottom of the channel and counteracts the drag force, creating a three dimensional trap.

(A)

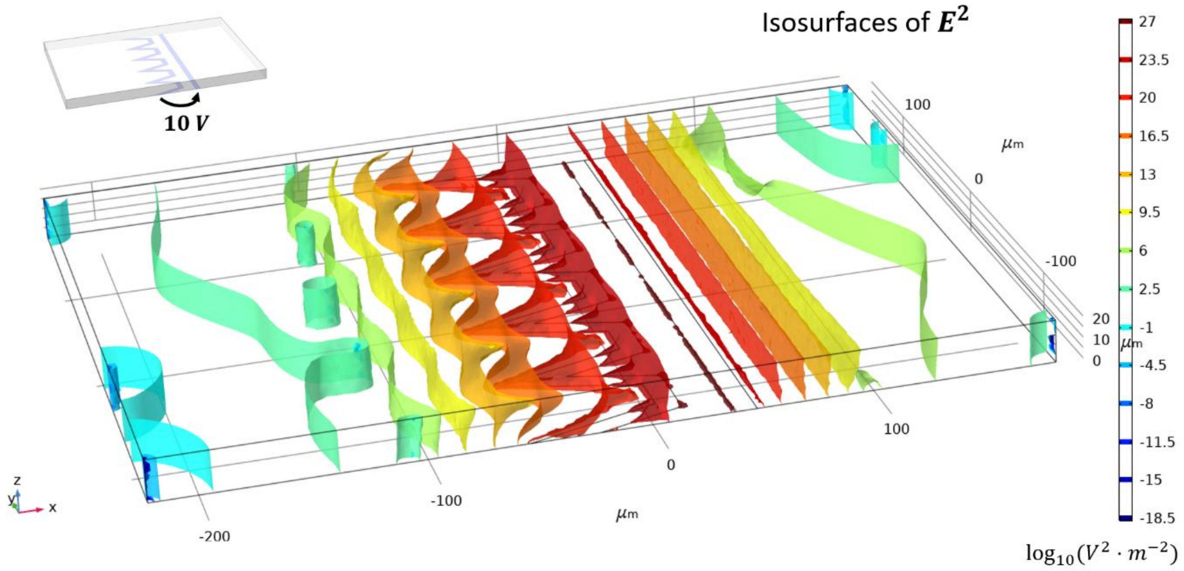

(B)

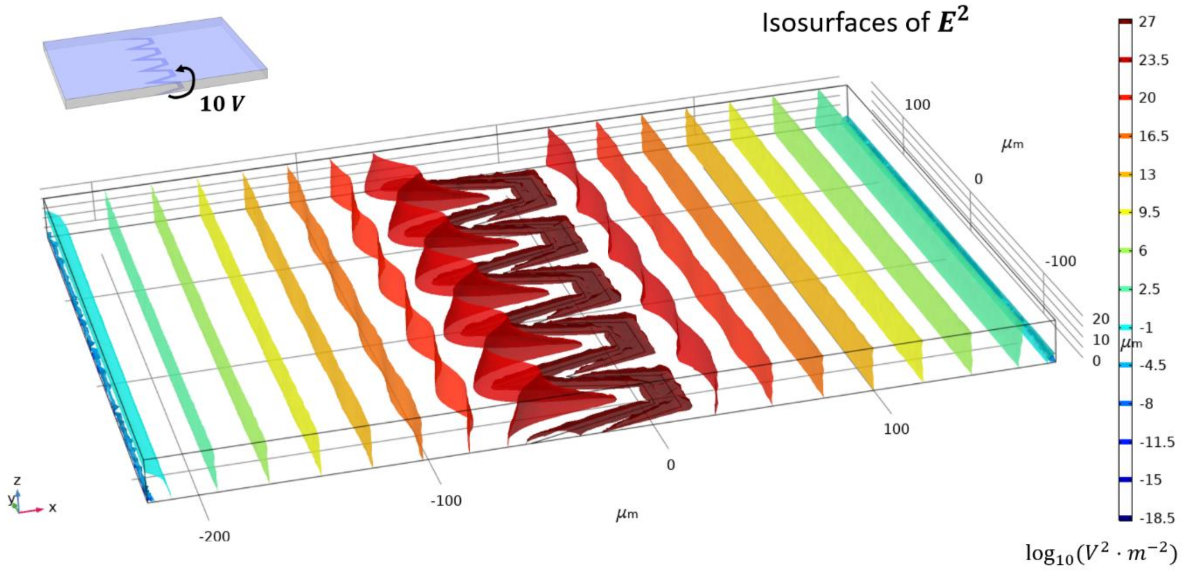

Figure S1 (A) Coplanar configuration (B) Facing configuration

Voltage dependency of the gradient term of the DEP force

Starting from the assumption that the expression of the potential φ in space is the factor of the voltage V applied to the electrodes and a function of space γ describing its distribution:

- $\varphi(\boldsymbol{r}) = V \cdot \gamma(\boldsymbol{r})$

The expression of the electric field $\boldsymbol{E}$ becomes:

$$\boldsymbol{E} = -\boldsymbol{\nabla}\varphi = -\boldsymbol{\nabla}[V \cdot \gamma(\boldsymbol{r})] = -V\boldsymbol{\nabla}[\gamma(\boldsymbol{r})]$$

We can thus rewrite

$$\boldsymbol{\nabla}|\boldsymbol{E}|^2 = \boldsymbol{\nabla}(\boldsymbol{E} \cdot \boldsymbol{E}) = \boldsymbol{\nabla}\big[\boldsymbol{\nabla}[V \cdot \gamma(\boldsymbol{r})] \cdot \boldsymbol{\nabla}[V \cdot \gamma(\boldsymbol{r})]\big] = V^2\ \boldsymbol{\nabla}[\boldsymbol{\nabla}\gamma(\boldsymbol{r}) \cdot \boldsymbol{\nabla}\gamma(\boldsymbol{r})]$$

Which becomes

$$\boldsymbol{\nabla}|\boldsymbol{E}|^2 = V^2 \cdot \boldsymbol{\alpha}(\boldsymbol{r})$$

With $\boldsymbol{\alpha}(\boldsymbol{r}) = \boldsymbol{\nabla}[\boldsymbol{\nabla}\gamma(\boldsymbol{r}) \cdot \boldsymbol{\nabla}\gamma(\boldsymbol{r})]$
